# Mineral accumulation, relative water content and gas exchange are the main physiological regulating mechanisms to cope with salt stress in barley

**DOI:** 10.1038/s41598-024-65967-5

**Published:** 2024-06-28

**Authors:** Faiza Boussora, Tebra Triki, Leila Bennani, Mohamed Bagues, Sihem Ben Ali, Ali Ferchichi, Kamel Ngaz, Ferdaous Guasmi

**Affiliations:** 1 Drylands and Oases Cropping Laboratory LACO, Institute of Arid Lands of Medenine (IRA), Sreet El Djorf 22.5 km, 4119 Medenine, Tunisia; 2 Department of Rural Engineering, Water, and Forests GREF, National Institute of Agronomic Research of Tunis (INAT), 43 Charles Nicolle, 1082 Tunis, Tunisia

**Keywords:** Barley, Salinity, Tolerant, Gas exchange, Biochemical traits, Physiology, Photosynthesis, Plant development, Plant physiology

## Abstract

Salinity has become a major environmental concern for agricultural lands, leading to decreased crop yields. Hence, plant biology experts aim to genetically improve barley’s adaptation to salinity stress by deeply studying the effects of salt stress and the responses of barley to this stress. In this context, our study aims to explore the variation in physiological and biochemical responses of five Tunisian spring barley genotypes to salt stress during the heading phase. Two salinity treatments were induced by using 100 mM NaCl (T1) and 250 mM NaCl (T2) in the irrigation water. Significant phenotypic variations were detected among the genotypes in response to salt stress. Plants exposed to 250 mM of NaCl showed an important decline in all studied physiological parameters namely, gas exchange, ions concentration and relative water content RWC. The observed decreases in concentrations ranged from, approximately, 6.64% to 40.76% for K^+^, 5.91% to 43.67% for Na^+^, 14.12% to 52.38% for Ca^2+^, and 15.22% to 38.48% for Mg^2+^ across the different genotypes and salt stress levels. However, under salinity conditions, proline and soluble sugars increased for all genotypes with an average increase of 1.6 times in proline concentrations and 1.4 times in soluble sugars concentration. Furthermore, MDA levels rose also for all genotypes, with the biggest rise in Lemsi genotype (114.27% of increase compared to control). Ardhaoui and Rihane showed higher photosynthetic activity compared to the other genotypes across all treatments. The stepwise regression approach identified potassium content, K^+^/Na^+^ ratio, relative water content, stomatal conductance and SPAD measurement as predominant traits for thousand kernel weight (R2 = 84.06), suggesting their significant role in alleviating salt stress in barley. Overall, at heading stage, salt accumulation in irrigated soils with saline water significantly influences the growth of barley by influencing gas exchange parameters, mineral composition and water content, in a genotype-dependent manner. These results will serve on elucidating the genetic mechanisms underlying these variations to facilitate targeted improvements in barley's tolerance to salt stress.

## Introduction

Salinization is intensifying over the earth's surface on a regular basis, it is getting amplified and intensified by climate change and anthropogenic practices, mainly irrigation. Indeed, this widespread phenomenon has already touched 400 million hectares of agriculture lands, and an equivalent area is menaced by this problem^1,2^. Thus, it represents a real threat to world food security.

Salt stress is considered a main limiting factor for plant growth and development and leads to significant reductions in crop productivity^3–5^. It dramatically influences the growth and productivity of plants resulting in several physiological disturbances. Salinity, as a primary stress, generates various secondary stresses mainly osmotic, ionic and oxidative stresses^6,7^. Osmotic effects show up in inhibition of water uptake by the root system which leads to limited water availability in cells. Consequently, plant cells experience water deficit, which affects cell turgor and leads to reduced cell expansion and growth. The limited water availability affects various physiological processes, including nutrient transport, photosynthesis, and metabolic activities, ultimately impacting plant health and productivity^8,9^. Ionic stress occurs when the content of cytosolic chloride in developed leaves is increased, causing, in turn, an increase in leaf senescence with chlorosis and necrosis^10^. Chlorosis occurs as a result of chlorophyll degradation and impaired photosynthetic activity, reducing the plant's ability to produce energy, Necrosis follows as cellular structures break down, leading to the death of leaf tissue^11^. Therefore, perturbation of water absorption and transport from the ground to the roots followed by a decline in the rate of shoot growth have been identified as the two primary consequences of these phenomena^8,11,12^. A decline in the performance of essential cellular metabolic pathways including photosynthesis is noticed. Therefore, studies that evaluate gas exchange and photosynthetic efficiency are important for the screening of salinity-tolerant plants.

Under salinity stress conditions, plants activate a wide range of adaptation processes in order to maintain cellular homeostasis. Sustaining the osmotic gradient by maintaining an optimal level of compatible osmolytes^13,14^ and regulating the balance between K^+^ and Na^+^ accumulation in different tissues are the main protective strategies to mitigate the detrimental effects of high salt concentrations^11,14^. These osmolytes include amino acids such as proline, notably. Plants often exhibit a significant accumulation of proline as a common physiological reaction to various environmental stresses^15^. To defend themselves, plants employ a protective strategy of excluding excess Na^+^ ions while maintaining high concentrations of K^+^ ions. Under extreme salinization conditions, plants limit the entry of Na^+^ or sequester this ion in their older tissues. This creates a "storage" environment, which can later be eliminated, thereby protecting the vital parts of the plant from salt-induced damage^16,17^. These strategies ensure a favourable intercellular K^+^/Na^+^ ratio and effectively regulates ion stability within plant cells^18^. Ions play also a crucial role in salt stress signalling. Cations such as Na^+^, Ca^2+^, Mg^2+^, and K^+^, along with anions like Cl^−^, SO_4_^2−^, HCO_2_^−^, CO_2_^2−^, and NO_2_^−^, interact to maintain cellular homeostasis. It is through the intricate balance of these ions that cell function is regulated under salt stress conditions^19^.

Sustaining an optimal gas exchange process is another key mechanism for enhancing plants tolerance to salinity. The uptake of carbon dioxide (CO_2_) and the release of oxygen (O_2_) through stomata are critical for photosynthesis and respiration. Efficient gas exchange ensures that plants maintain the necessary CO_2_ levels for photosynthesis, which is essential for energy production and growth^17,20^. Under salt stress, plants regulate the opening and closing of stomata to balance CO_2_ uptake with water loss, preventing excessive water loss while ensuring adequate CO_2_ for photosynthesis. This strategy ensures also efficient respiration for energy production. Furthermore, efficient gas exchange can help mitigate oxidative stress by regulating the balance of O_2_ and CO_2_, thus protecting cells from damage caused by reactive oxygen species (ROS)^21,22^. However, when plants experience salt stress, a high accumulation of Na^+^ in the cytoplasm leads to stomatal closure. This closure triggers an imbalance between the absorption of light through photosystem II (PS II) and the utilization of energy. Consequently, this disruption results in a reduction in the photosynthesis rate, interferes with the bio-energetic processes inherent to photosynthesis, and gives rise to the production of reactive oxygen species (ROS)^23–26^.

Hence, addressing the problems of salinity, which has an impact on plant growth and limits agricultural production around the world, will be necessary in the future, given the growing population of the planet and the ever-increasing demand for food^27^. Among cereals, Barley (*Hordeum vulgare* L.) is regarded as the most salt-resistant species^28,29^. According to the FAO's report, the worldwide average seed production and harvested area for barley were approximately 3.50 metric tons per hectare and 504,000 hectares, respectively, in 2020^30^. In fact, this crop is well-suited for arid areas where precipitation is rare and soils and water are saline. These conditions are typical in southern Tunisia, where barley is the most cultivated cereal^31,32^. Hence, the resilience of barley to salinity and drought stress provides valuable insights that can be applied to improve the cultivation of other crops under similar conditions. In addition to this feature, Barley grains offer various health advantages owing to their mineral composition, protein content, fiber, and diverse vitamin content, thus it is integral to the production of traditional foods and beverages, contributing to the local economy and cultural heritage^33^. In addition to its use in human nutrition, barley is also used as animal feed, which supports the livestock industry. Hence, this valuable crop holds significant economic and industrial importance in Tunisia, particularly in the southern regions where it is the most cultivated cereal. It is a crucial crop for local agriculture due to its adaptability to arid climates and saline soils, making it a reliable source of income for farmers in these challenging environments^34,35^. However, in our previous study^32^, we showed that salinity had a direct effect on spike growth and spike development and through this an indirect effect on plant yield. Studies have shown also that salinity decreases relative water content (RWC), and alters nutrient uptake^6^. Research also indicated that salt stress can affect the hormonal balance within barley plants, leading to altered levels of growth regulators such as abscisic acid^32,36^. In the current study, we hypothesized that different barley genotypes exhibit varying levels of salt tolerance due to their differential ion accumulation and osmotic adjustment capabilities. Therefore, identifying response mechanisms is needed to assess for salt tolerant genotypes within this species, to develop strategies to enhance barley's salt tolerance and to ensure sustainable production in saline environments. The main aims of the current study are to screen the effects of salt stress on barley plants on heading stage and to identify most tolerant barley genotypes based on a set of physiological, biochemical, and ionic traits.

## Results

### Physiological stress parameters

**Figure 1:**
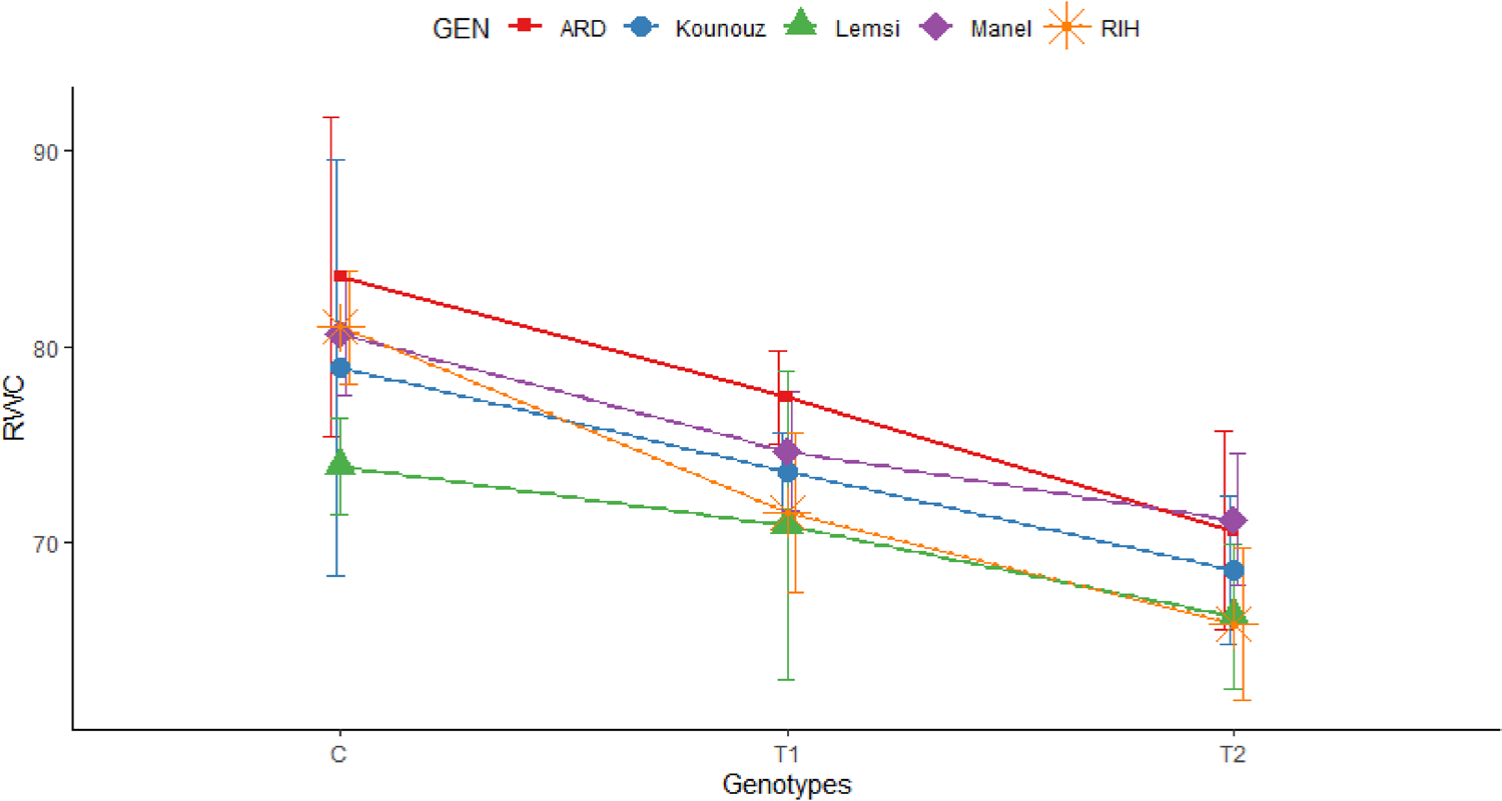
Relative water content (%) of five barley genotypes under control and salt stress. C: Control; T1: 100 mM NaCl; T2: 250 mM NaCl.

**Figure 2:**
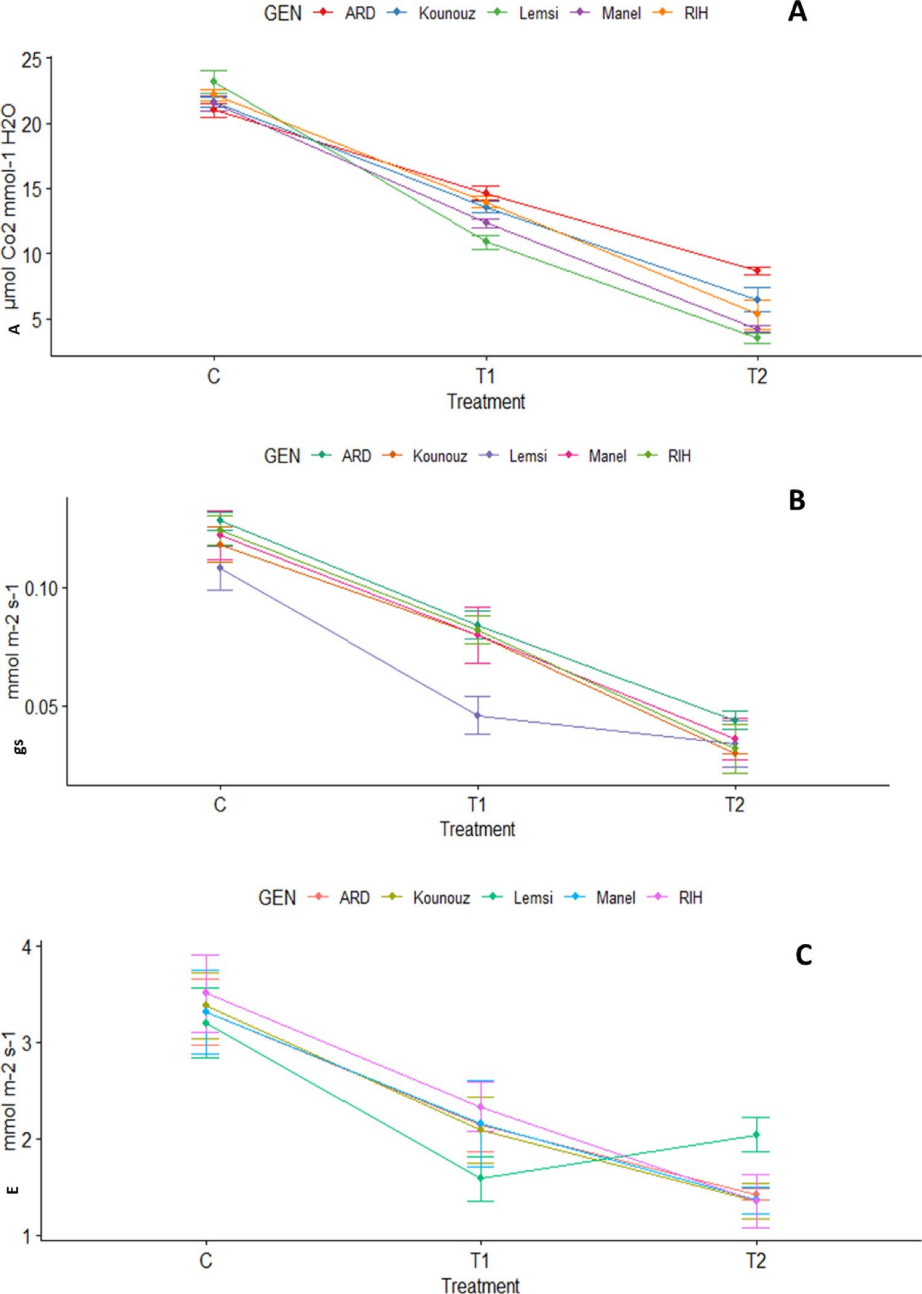
Gas exchange traits (photosynthetic rate, (**A**); transpiration rate, (**B**); and stomatal conductance, (**C**)) of studied barley genotypes under control and salinity stress. C: Control; T1: 100 mM NaCl; T2: 250 mM NaCl.

Salinity stress led to a substantial decrease in the relative water content (RWC) and gas exchange parameters, namely, photosynthesis rate (A), transcription (E) and stomal conductance (gs) in all studied barley genotypes. Measurements were conducted during the heading stage, and the results are depicted in Figs. 1 and 2.

Under control conditions, all five barley genotypes exhibited the highest relative water content RWC, however, when subjected to salt stress treatments, reductions in RWC were observed compared to the control. the reduction is more pronounced when plants have been exposed to 250 mM of NaCl. Ardhaoui genotype showed a 6.12% reduction at 100mM NaCl and 12.90% at 250 mM NaCl. Results showed also that Lemsi genotype had the smallest reductions, with 3.02% at 100mM NaCl and 7.65% at 250 mM NaCl, indicating the highest salt tolerance. While Rihane genotype presented the highest sensitivity to salt stress with a 9.39% reduction at 100mM NaCl and 15.09% at 250 mM NaCl. Tour results highlight Lemsi as the most resilient genotype and Rihane as the most sensitive genotype under saline conditions.

We also observed a decrease in photosynthetic activity across all examined genotypes (Fig. 2), concomitant with diminished stomatal conductance and transpiration within the same genotypes. The analysis of variance shows a notably significant impact of salty stress on all photosynthetic parameters, including CO_2_ assimilation rate (A), stomatal conductance (gs), and transpiration (E). Genotype-related distinctions and interactions between treatments and genotypes are Significantly evident across all parameters except stomatal conductance and transpiration.

**Figure 3:**
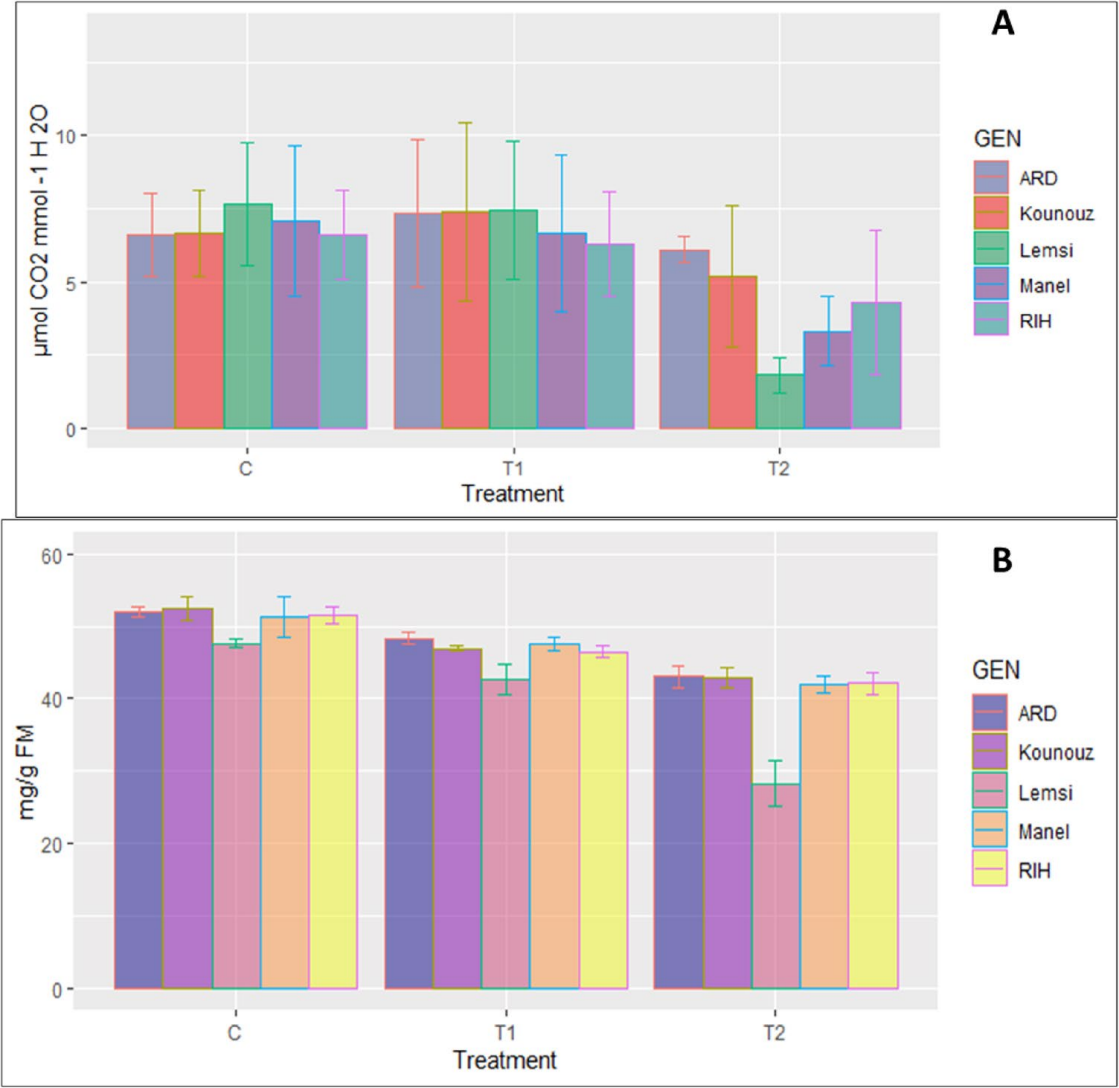
The impact of salt stress on Water Use Efficiency (WUE): (**A**) and SPAD: (**B**) of five barley genotypes under control and salinity stress. C: Control; T1: 100 mM NaCl; T2: 250 mM NaCl.

The Fig. 3 illustrates the effect of salt stress on the water use efficiency (WUE) in salinized and non-salinized barley plants and the large diversity among genotypes. It is evident that salt stress exerts a pronounced influence on WUE levels across all genotypes, highlighting statistically significant differences in WUE levels between the tested genotypes. Of particular interest Lemsi is showing notably lower WUE recorded values in comparison to the other genotypes These distinctions indicate that certain genotypes exhibit a superior ability to maintain WUE under salt stress, while others are more susceptible to its detrimental effects.

### Ion concentrations

**Figure 4:**
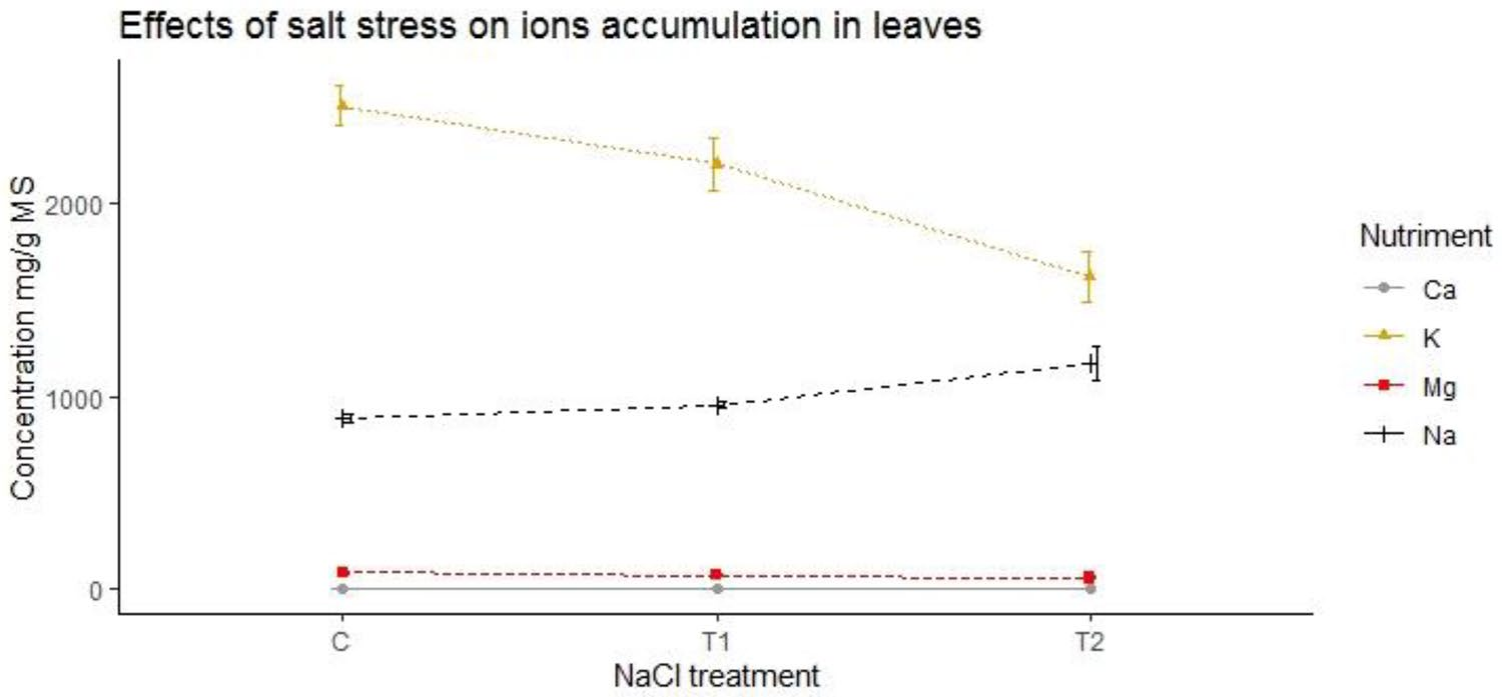
The effect of salt stress on ions accumulation of studied barley genotypes under control and saline stress C: Control; T1: 100 mM NaCl; T2: 250 mM NaCl.

This study examined the mineral content of five genotypes of barley under salinity stress. Results illustrated in Fig. 4 showed a general reduction in the concentration of K^+^, Ca^2+^, and Mg^2+^ ions, with higher accumulation levels in non-stressed leaves. All genotypes showed a decline in their potassium content, with inter-genotypic disparities arising due to saline stress. Ardhaoui showed a notable decrease in potassium content, with a reduction of 32.30% at the highest salt concentration, conversely, Lemsi exhibited a substantial increase in sodium content, with an increase of 43.67% at 250 mM NaCl, indicating its high capacity for sodium accumulation under severe salt stress. Additionally, Manel showed a pronounced decrease in calcium levels, with a reduction of 42.35% at the highest salt concentration. These key findings illustrate the differential ion regulation mechanisms employed by various barley genotypes in response to salinity, with some genotypes like Lemsi displaying higher tolerance through sodium accumulation, while others like Ardhaoui and Manel are more adversely affected by the loss of essential ions. Additionally, saline stress conducted to a reduction in Mg^2+^ ion content within leaves of all genotypes by 10% at a NaCl concentration of 100 mM and by 20% at a concentration of 250 mM NaCl (Fig. 4). These findings suggest that stress-induced reduction in K^+^ ion content is less pronounced in resistant genotypes. Saline stress led to a significant reduction in foliar Ca^2+^ content across all genotypes, particularly affecting Ardhaoui genotype, which displayed heightened sensitivity to saline stress (Fig. 4). The differential responses among genotypes were highly significant for sodium and potassium, respectively.

**Figure 5:**
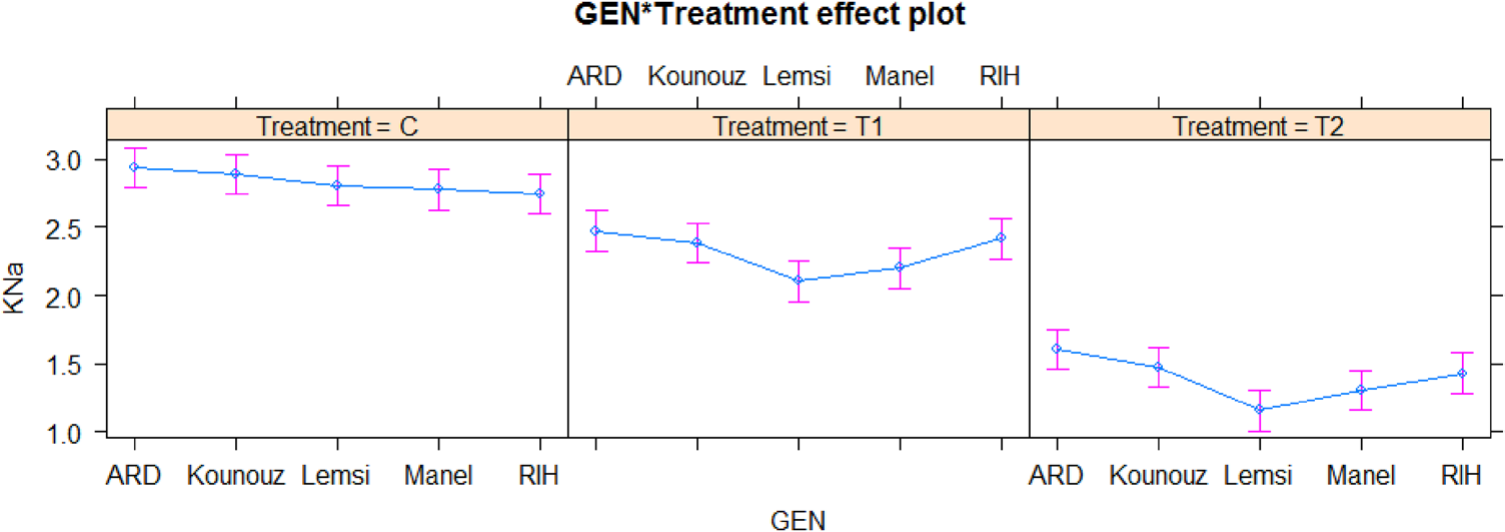
The effect of saline stress on K/Na ratio of five studied barley genotypes under control and salt stress. C: Control; T1: 100 mM NaCl; T2: 250 mM NaCl.

The K^+^/Na^+^ ratio is is a significant trait to identify salinity tolerance and it was noted that all barley genotypes showed a gradual decrease below saline stress compared to control. However, the maximum decline was observed in salt-sensitive genotype Lemsi. This decline was intense when plants were exposed to 250 mM NaCl as given in Fig. 5.

### Biochemical stress parameters

**Figure 6:**
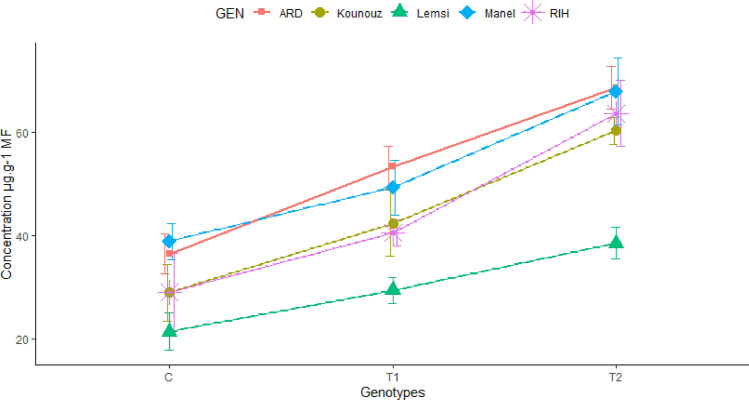
Proline content in leaves of studied Barley genotypes under salt stress with 100 and 250 mM NaCl.

Under salt stress conditions, the proline content in barley genotypes showed significant increases. Upon analyzing our results, we noticed an increase in proline content of an average of 1.39 times for the entire of genotypes treated with 100 mM NaCl in comparison with the control and an average of 1.94 times for the entire genotypes treated with 250 mM NaCl in comparison with the control. Rihane genotype showed the most remarkable increase, with a proline accumulation of 2.19 times the control at the highest salt concentration (250 mM NaCl). Similarly, Kounouz displayed a substantial increase, with proline levels rising to 2.08 times the control under the same conditions. These increases indicate that Rihane and Kounouz have a high capacity for proline accumulation in response to severe salt stress, suggesting a robust mechanism for osmotic adjustment and stress tolerance. Ardhaoui genotype also showed a notable increase, though slightly lower, at 1.88 times the control (Fig. 6).

### Soluble sugars content

**Figure 7:**
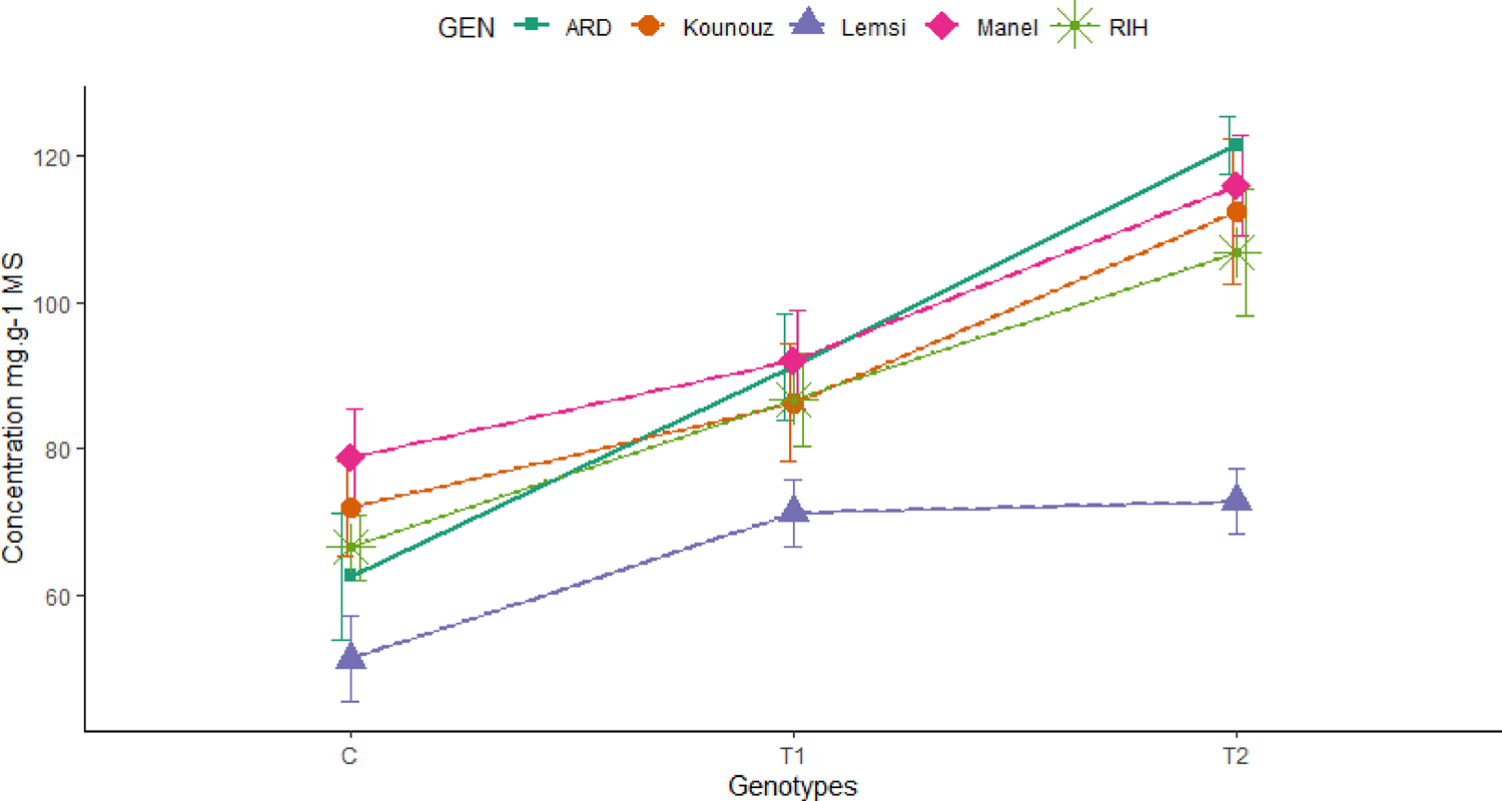
Soluble sugars content inside leaves of studied barley genotypes under saline stress with 100 and 250 mM NaCl.

Soluble sugars content was used also to differentiate the resistance of genotypes to salt stress. Under control conditions, genotypes have variable levels of soluble sugars ranging from 51.24 mg/g DW for Lemsi genotype to 78.8 mg/g DW for Manel genotype (Fig. 7). Under salt conditions, Ardhaoui genotype showed the most remarkable increase, with soluble sugar levels rising to 1.94 times the control at the highest salt concentration (250 mM NaCl) (Fig. 7). This indicates a strong stress response and an effective osmotic adjustment mechanism. Kounouz also displayed a substantial increase, with soluble sugar levels increasing to 1.56 times the control under the same conditions. Additionally, Rihane showed a notable rise, with an increase to 1.60 times the control. These increases suggest that Ardhaoui, Kounouz, and Rihane have a higher capacity for soluble sugar accumulation, enhancing their tolerance to severe salt stress. In contrast, Manel and Lemsi exhibited lower increases, with their soluble sugar content rising to 1.47 and 1.42 times the control, respectively, under the highest salt stress condition, indicating a comparatively less robust response to salinity (Fig. 7).

These sugars are believed to be responsible for maintaining the integrity of membrane structures, particularly chloroplasts and mitochondria, which would imply better plant adaptation to stress. This is the case for Ardhaoui genotype, which appears to increase its soluble sugar content almost twice as much as the control at 250 mM NaCl.

### Malondialdehyde (MDA) content

Malondialdehyde (MDA) is one of the end products resulting from peroxidation of lipids that reflects the presence of stress. Therefore, it is considered to be an excellent marker of plant tolerance to various abiotic stresses^37^. Since MDA measurement informs about the state of degradation of cell membranes, we have examined the lipid peroxidation through the measurement of MDA content in the five studied barley genotypes under the effect of salt-induced salinity stress.

**Figure 8:**
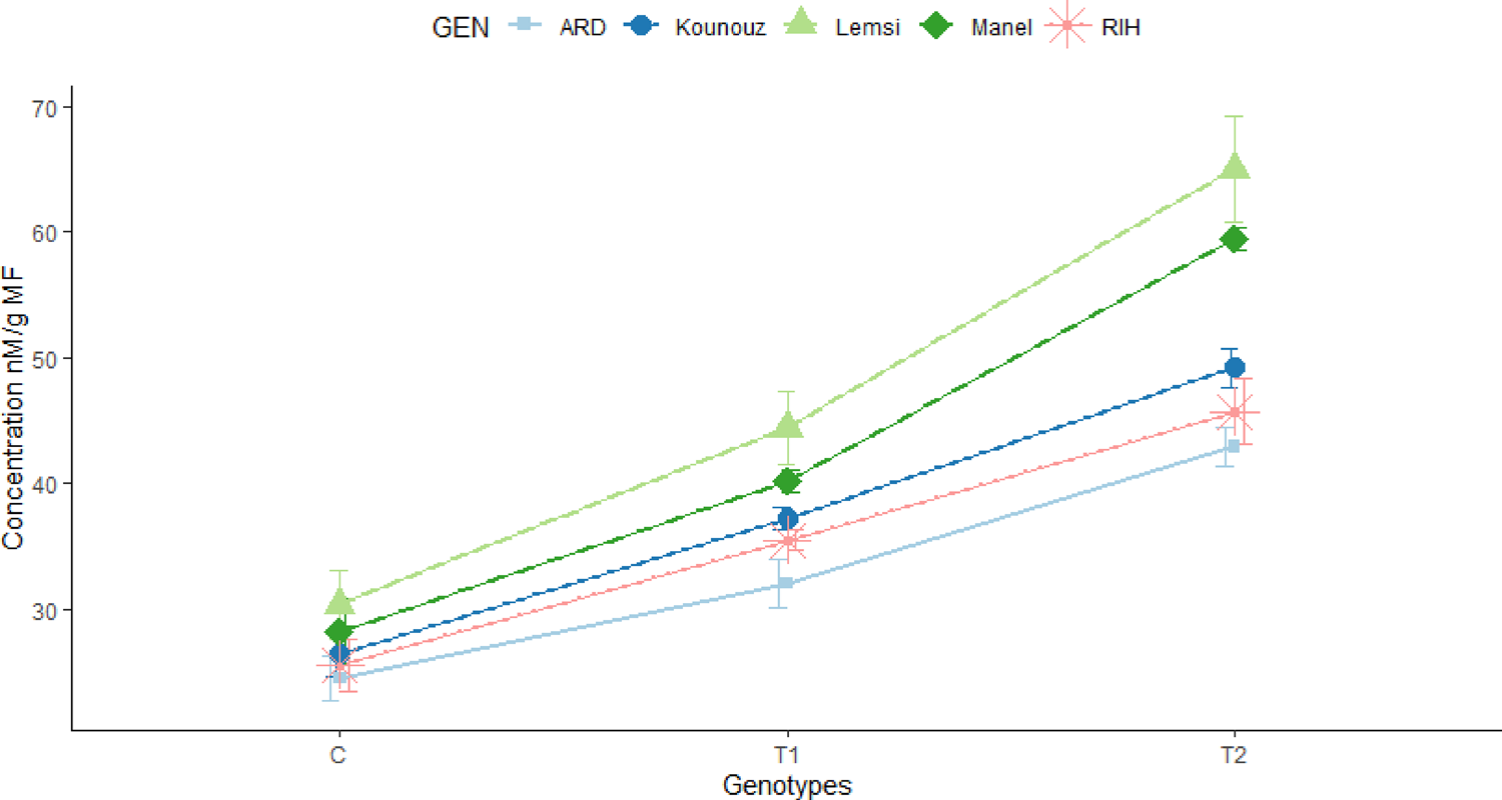
MDA content into leaves of studied Barley genotypes under salinity stress with 100 and 250 mM NaCl..

In the investigation of MDA levels among different genotypes subjected to salt stress, Lemsi emerged as the most sensitive genotype, exhibiting the highest MDA level of 30.35 nM/g MF, which dramatically surged to 44.43 nM/g MF under 100 mM NaCl treatment, marking a substantial 46.37% increase compared to the control. Impressively, under 250 mM NaCl treatment, Lemsi recorded the highest MDA level of 65.05 nM/g MF, indicating a striking 114.27% increase from the control. Following closely, Kounouz demonstrated notable increases in MDA levels, with a baseline of 26.37 nM/g MF rising to 37.27 nM/g MF under 100 mM NaCl treatment and further escalating to 49.21 nM/g MF under 250 mM /L NaCl treatment, marking significant increases of 41.31% and 86.57%, respectively. Similarly, Manel and Rihane also showed considerable increases in MDA levels under salt stress, suggesting varying degrees of susceptibility among different genotypes. (Fig. 8).

### Multivariate analysis

**Figure 9:**
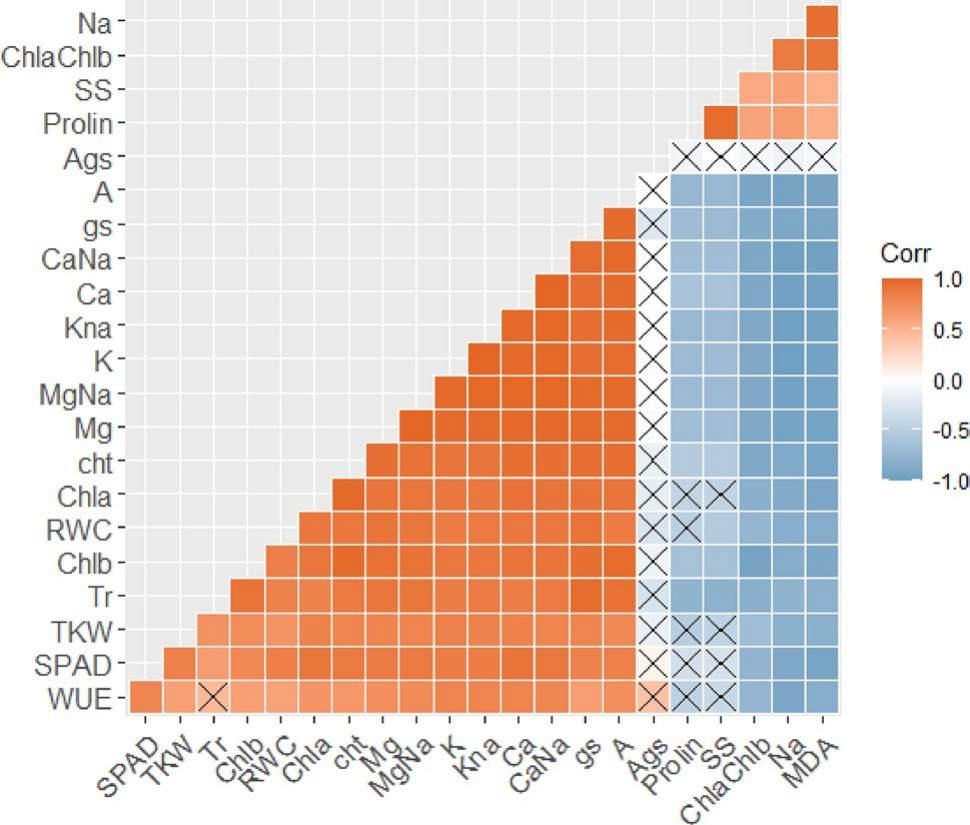
Pearson correlation amongst all the studied traits below salt conditions. The color of the matrix grid indicates the strength of correlation between the traits and genotypes. The altitude of correlation is indicated by orange for positive correlation, blue for negative correlation, and white for the lack of correlation.

Thousand kernel weight (TKW) per plant was positively and significantly (p ≤ 0.001) correlated with photosynthesis (A), transpiration (E), Stomatal conductance (gs), water use efficiency (WUE), relative water content (RWC), and ions (K^+^, Ca^2+^ and Mg^2+^) under salinity stress circumstances. However, proline, MDA and SS showed a negative and significant correlation with TKW under the same salinity stress circumstances (Fig. 9).

**Figure 10:**
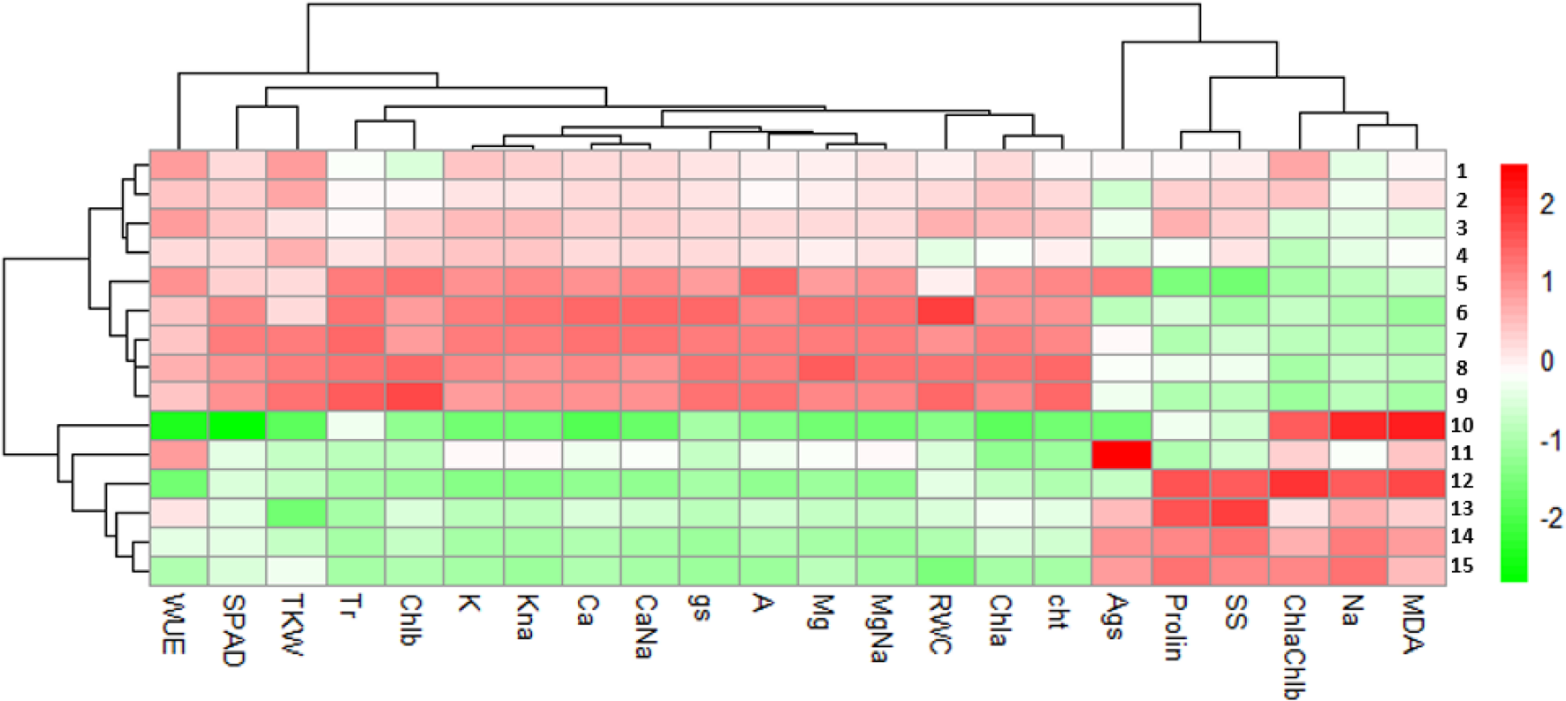
Diagram and hierarchical clustering analysis by Euclidian distance using Ward’s method generated from means value of physiological and biochemical traits of five barley millet genotypes. The color of the heat map grid designates the force of correlation between the traits and genotypes. The correlation levels are indicated by red for positive correlation, green for negative correlation, and white for the lack of correlation, as demonstrated in the color key at top left. The package ‘pheatmap’ was employed for hierarchical clustering, and Euclidean distances were employed to calculate the distance matrix.

The heatmap (Fig. 10) showed the clustering of the five barley genotypes, established by the means of their biochemical and physiological values, into two distinct groups. The first group could be further divided into two sub-groups. The first sub-group comprises four genotypes (Ardhaoui, Kounouz, Lemsi and Manel) under control conditions. The second sub-group contains five genotypes; Ardhaoui, Kounouz, Lemsi and Manel under 100 mM of NaCl with Rihane under control conditions. The second group contained five genotypes; Ardhaoui, Kounouz, Lemsi, Manel and Rihane under 250 mM conditions and Rihane under 100 mM of NaCl conditions. We can observe a clear segregation based on the physiological traits.

### Barley traits priority under salinity stress

To identify the effect of component variables on thousand kernel weight (dependent variable), all possible and stepwise regression analyses were assessed. All possible regression analyses indicated that potassium content, K^+^/Na^+^ ratio, relative water content, stomatal conductance and SPAD measurement contributed significantly to thousand kernel weight of barley under salt stress while the remaining traits contribution was non-significant to grain crop (Table S2; F27-31). Then, we removed the non-significant characters during the stepwise regression method. Obtained results showed that potassium content, K^+^/Na^+^ ratio and relative water content communally reported more than 70% of the thousand kernel weight distinction under salinity stress. Further, potassium content, K^+^/Na^+^ ratio, relative water content, conductance of stomata and SPAD measurement with cumulative R2 = 84.06 played a significant role in the thousand kernel weight variation (Table 1) as well as might be best fixed since it revealed the least Mallows’ Cp criterion. Using the regression coefficients of respective characters, the subsequent equation was calculated to predict the expected barley thousand kernel weight below salty stress (Table S2; F27-31).

$$\begin{aligned} {\text{Predicted barley thousand kernel weight}} &= - {3}.{99}\, + \,\left( {0.0{78}\, \times \,{\text{K}}^{ + } } \right)\, + \,\left( { - { 53}.{43}\, \times \,{\text{K}}^{ + }/{\text{Na}}^{ + } } \right)\, \hfill \\ & \quad + \,\left( { - 0.{\text{96 RWC}}} \right)\, + \,\left( {{261}.0{1}\, \times \,{\text{gs}}} \right)\, + \,(0.{844}\, \times \,{\text{SPAD}}). \hfill \\ \end{aligned}$$

**Table 1:** Traits modelling for salt tolerance though various linear regressions method.

Dependent variable	Step and variables	Cp	R-square
TKW	1. K^+^	28.37	68.57
	2. K^+^ + K^+^/Na^+^	15.32	71.86
	3. K^+^ + K^+^/Na^+^ + RWC	9.40	71.94
	4. K^+^ + K^+^/Na^+^ + RWC + gs	7.72	75.53
	5. K^+^ + K^+^/Na^+^ + RWC + gs + SPAD	9.49	84.06

Mallows’ Cp Criterion is a manner to evaluate the fit of a multiple regression model; minor Cp values are better as they show smaller amounts of unexplained error.

## Discussion

The salinity of soil and nutrient deficiency are major growing problems that affect agricultural lands worldwide^38^. Gaining insight into the response of model plant yields, namely barley’s response to salinity stress is a crucial stride in the direction of creating salt-tolerant yield varieties that can contribute mostly to reclamation and long-term agricultural usage of affected lands. Salinity stress unfavourably influences the progress and developmental characters in barley by diminishing the number of tillers, plant height, root length, shoot length, fresh and dry root weight, and plantlet weights which eventually reduced the yield of barley^32,39^. In this work, as expected, salinity stress (100 mM and 250 mM) decreased the physiological traits namely, gas exchange parameters and mineral nutrition in all studied barley genotypes. When salinity reached 250 mM NaCl, significant changes were observed in relative water content (RWC) and malondialdehyde (MDA) levels, indicating a substantial impact on barley physiology. The decrease in RWC suggests that the plants experienced severe water stress, likely due to the osmotic effects of high salt concentrations, which reduce water uptake and availability^9,40^. This water deficit can impair various physiological processes, leading to reduced cell turgor and impaired metabolic functions and causing a decrease in WUE, water uptake capability, leaf water potential, and transpiration rate under salt stress^6,9,41^. Previous studies demonstrated that the higher the foliar relative water content, the more tolerant the genotype is to stress^42,43^. Similarly, Ma et al.^44^ observed that cotton plants respond to saline stress by reducing their foliar relative water content, and this reduction is particularly pronounced when the stress is severe. Lemsi genotype was capable to keep the highest water content compared to the studied genotypes, which might be accredited to its ability to delay stress. According to^6,42,45^, tolerant barley genotypes are better able to maintain their foliar water status than sensitive ones. Likewise^8^, working on wheat, found that the tolerant variety preserved a very important relative water content associated to the sensitive variety, and therefore, it was able to maintain a high foliar water potential.

Simultaneously, the increase in MDA levels under 100 mM NaCl and 250 mM NaCl in all studied barley genotypes highlights oxidative stress within the plants and indicates the critical impacts of salt. In fact, MDA is a byproduct of lipid peroxidation, a process that occurs when reactive oxygen species (ROS) damage cell membranes and plant tissues, compromising membrane integrity and function^9,46^. Lipid membrane peroxidation is associated with a malfunction of the detoxification system, which could lead to damage of the main cellular components^41^. Our results are consistent with^9,47^, who showed that MDA content increases under salt stress in barley. Horchani et al.^46^ also reported that under severe salt stress, sensitive barley genotypes accumulate more MDA than tolerant genotypes, as in the case of Ardhaoui genotype compared to Lemsi genotype. Increased oxidative damage can severely impact plant growth, photosynthetic efficiency, and overall health, in fact, negative effects due to salinity have been detected for net photosynthesis (A), transpiration rate (E), and stomatal conductance (gs) in all barley genotypes. The reduction in photosynthetic activity in plants under salinity stress is cited by several authors as one of the main origins of reduced plant development and production^6,48,49^. Indeed, plants exposed to salinity are subjected at the same time to water insufficiency and ion stress. According to several authors, to cope with the effects of drought, the plant tries to limit water loss by reducing the stomatal aperture. Nevertheless, extended stomatal closure has a negative effect on photosynthetic activity and consequently on plant development and productivity^22,42^. The reductions in gs and E could be a response of the plant to the decline in water potential of the environment. Our results are coherent with those of^6,10^, who have also demonstrated that salt stress significantly reduces different gas exchange characteristics such as A, E and gs in barley plants. Under these conditions, there's an observed increase in water use efficiency (WUE), which is demonstrated by a robust positive correlation between stomatal conductance (gs) and photosynthetic activity (A). This correlation serves as a mechanism of adaptation to stress in these plants. Several studies have consistently pointed towards an enhancement in water use efficiency (WUE) under salt stress, primarily due to a significant reduction in transpiration relative to photosynthetic activity. This adjustment allows plants to optimize water usage in stressful environments, contributing to their survival and growth under challenging conditions^7,50,51^.

Similar to physiological traits, ionic concentrations of barley leaves were changed due to salinity stress. One of the plant’s defence mechanisms to cope with the harmful effects of excessive Na^+^ is balancing between Na^+^ and K^+^ accumulation in different plant tissues^16,46^. Based on obtained results, salinity stress significantly declined the means of the content of K^+^ in shoot tissues as well as the K^+^/Na^+^ ratio across all the evaluated barley genotypes. A general decrease in the concentration of Ca^2+^ and Mg^2+^ ions was also detected. This indicates that K^+^ absorption is particularly sensitive to stress. Vacuolar exchange selectivity of K^+^/Na^+^ seems to be decisive in tolerance to salinity in certain species such as barley and wheat^41,46^. Reacting to salt, the reduction in potassium content in different barley genotypes might be caused by the transfer of these ions from leaves to roots to increase the osmotic potential in root cells^41,52^. Researches have revealed that in barley, K^+^ is very abundant compared to divalent cations, it has an essential function in photosynthesis and represents one of the ions involved in osmotic regulation^6,53^. However, the function of calcium in improving tolerance to salinity is attributed to calcium's intervention in maintaining the integrity of foliar and root cell membranes and in ionic selectivity^9,54^. Indeed, the lack of calcium can guide to membrane structure disruption by degradation of the phospholipids that constitute it. It appears that varieties with the greatest ability to accumulate calcium are those that will exhibit better membrane stability^9,16,54^. According to our outcomes, the genotypes Ardhaoui and Rihane showed a discrete decrease in their Ca2^+^ content in comparison to Lemsi genotype. Therefore, plant tolerance to salt may be owed to the maintenance of a high Ca^2+^ content within the leaves, which would probably serve as protection for the apoplastic space or the surface of the plasma membrane. Calcium also appears to significantly promote K^+^ absorption. Therefore, the diminution in calcium in barley genotypes could interfere with potassium absorption. This nutritional imbalance, when essential ions such as Ca^2+^, K^+^ or Mg^2+^ become limiting, is probable to affect growth by limiting cell expansion and inhibiting the photosynthetic process^16,18,21^. Indeed, within the plant, K^+^ and Mg^2+^ have important, identical or distinct functions regarding water status, including regulating stomatal opening and closing, plant osmotic adjustment and root growth^18,55^. Many studies suggest that the increased sodium ion Na^+^ absorption in saline conditions resulted from compromised water absorption and decreased relative water content^18,46^.

To cope with the critical decrease in water availability, barley plants accumulate several osmolytes such as proline and various sugars, these osmolytes play crucial roles in osmotic adjustment, helping to maintain cell turgor and protect cellular structures under stress conditions^14,56^. The accumulation of proline is frequently observed in several species under the influence of different environmental stresses^57,58^. This particular amino acid, derived from the synthesis of glutamic acid, has the ability to serve as a regulatory or signalling molecule, prompting various responses essential to the adaptation process^14,58,59^. Under stress, all genotypes respond with an accumulation of proline that varies depending on the genotype in question. This increase in proline content seems to be related to barley's response to osmotic stress. studies have also shown that proline accumulation is related to stress tolerance and is not a consequence of tissue dehydration or reaction to damage caused by stress^14,58^. Despite the accumulation of proline, Ardhaoui genotype recorded a high decrease in relative water content under salt stress. This means that osmotic adjustment through proline accumulation is not the most effective pathway for maintaining leaf water potential in this genotype. Lemsi, Kounouz, and Rihane genotypes showed a low decrease in their relative water content, this moderate decrease was accompanied by a significant accumulation of proline, explaining that these genotypes adjust their water potential through an effective osmoregulation mechanism. Many researchers suggested that significant proline accumulation is positively correlated with water potential, which in turn is correlated with stress tolerance^17,60^. Beside proline, barley plants used also soluble sugars to alleviate the intensity of salt stress^61^. Actually, soluble sugars play a dual role in stressed plants, as they participate in metabolic events and dehydration, which is crucial in osmotic adjustment and the stabilization of certain proteins^9,42,61^. The accumulation of sugars appears to induce the gelation of cellular contents by saturating the intracellular environment; this phenomenon helps to prevent the crystallization of molecules contained in the cell, thus limiting damage to cellular structures^62^. The accumulation of sugars is suggested as an indicator of resistance to salt stress, in fact, several studies have also shown an accumulation of sugars in plant leaves during salt stress^50,63^. In the present study, the levels of soluble sugars showed an accumulation in all genotypes studied under saline stress conditions. This accumulation in the leaves varied differently among the genotypes studied with response to salinity. The accumulation of sugars is just an adaptation phenomenon to water stress, which allows the plant to maintain its turgor by reducing and adjusting the water potential^9,50,64^. In case of water deficiency, the accumulation of soluble sugars can be attributed to the hydrolysis of starch^62,65,66^ and the inhibition of certain metabolic pathways of synthesis. These sugars would be responsible for maintaining the integrity of membrane structures, particularly chloroplasts and mitochondria^62,67^, which would imply a better tolerance of the plant to water stress. This is the case for Ardhaoui and Rihane genotypes, which recorded the highest accumulations in soluble sugars.

## Material and methods

### Plant material and growing conditions

Based on their value and yield under stress conditions, five six-rowed spring barley genotypes Ardhaoui, Kounouz, Lemsi, Manel, and Rihane (Table S1) kept in the gene bank of the Arid Land Institue of Medenine Tunisia were chosen for this study. Barley seeds were sowed in the greenhouse of the Experimental Station of the same institute, (latitude 33o61′22"N, longitude 10o23′47″E), under long day circumstances of 16 h of daylight and 8 h of darkness, with temperatures of 20 °C during the day and 16 °C at night. Three sets of 96 seeds per genotype were planted in 96-well plates and allowed to germinate. One set was given tap water as a control, and the two other sets were given saline water (100 mM NaCl and 250 mM NaCl) commencing on the day of planting. Seedlings were transplanted into 14 cm diameter pots when they had three leaves, and they were watered with either tap water or saline water. The recommended agricultural procedures, such as controlling weeds, diseases, and pests, were followed. The leaf samples used for physiological and biochemical measurements were randomly collected from the control or salt treated during heading stage. All measurements were replicated three times with independent plant samples.

### Physiological assay

#### Relative water content

The relative water content (RWC) was determined according to the approach proposed by^68^. Briefly, 10 leaves from barley were weighed immediately (FW) after harvesting. The leaves were subsequently immersed in distilled water for a duration of 4 h, following which their turgid weight (TW) was quantified. Subsequently, the leaves underwent drying in an oven set at 80 °C for 24 h to determine their dry weight (DW). The RWC was calculated as follow:

$${\text{RWC }}\left( \% \right) \, = \, \left[ {\left( {{\text{FW}}{-}{\text{DW}}} \right)/\left( {{\text{TW}}{-}{\text{DW}}} \right)} \right] \, \times { 1}00.$$

### Gas exchange, photosynthetic pigments, and SPAD analysis

Gas exchange parameters such as photosynthetic rate (A), transpiration rate (E), stomatal conductance (gs), and water use efficiency (WUE) were measured using a portable gas-exchange system (ADC BioScientific LC ProSystem Serial No. 3302) on the flag leaves. The experimental conditions included a leaf temperature of 25 °C, an incident light intensity of 800 μmol photons m/s generated by a red/blue light source, and a controlled CO_2_ concentration of 400 μmol/mol. The leaf-to-air Vapor Pressure Deficit (VPD) was upheld at 1 kPa. Extraction and quantification of Chlorophyll a and Chlorophyll b were performed using a altered method of^69^. After the extraction and analysis, the relative amount of Chlorophyll a and Chlorophyll b were calculated using the following formulae:

$${\text{Chlorophyll a }}\left( {{\text{mg}}/{\text{g}}} \right) \, = \, \left[ {\left( {{12}.{7} \times {\text{A663 }} - { 2}.{69} \times {\text{A647}}} \right){\text{ V}}/{\text{W}}} \right]$$

$${\text{Chlorophyll b }}\left( {{\text{mg}}/{\text{g}}} \right) \, = \, \left[ {\left( {{22}.{9} \times {\text{A647 }} - { 4}.{68} \times {\text{A663}}} \right){\text{ V}}/{\text{W}}} \right].$$

Chlorophyll content (SPAD) was evaluated using a chlorophyll meter Type (Minolta 1500) at a rate of 6 repetitions per treatment.

### Mineral analysis

The mineral analysis was assessed referring to the method described by^70^. 1 g of finely ground plant material underwent combustion in a muffle furnace at 530 °C for a duration of 5 h. The resultant ash was dissolved by adding 5 mL of hydrochloric acid (20%), and the volume of the resulting solution was adjusted to 50 mL using distilled water in a volumetric flask. Subsequently, individual mineral elements were analyzed separately using an atomic absorption photometer (Shimadzu A 6800, Kyoto, Japan).

### Biochemical assay

#### Proline measurement

The content of proline was performed using to the method outlined by^71^. 100 mg of frozen leaves were ground. 1.8 ml of 3% sulfosalicylic acid has been added, the solution was then centrifuged at 14,000 rpm for 10 min. 1.5 ml of the supernatant from each sample is extracted and transferred to new screw-cap glass tubes. To this, 1.5 ml of acetic acid and 1.5 ml of ninhydrin solution containing 1.25 g of ninhydrin, 20 ml of 6M ortho-phosphoric acid, and 30 ml of acetic acid are added. This mixture is heated to boiling (100 °C) in a water bath for 1 h. The solution gradually turns red. After cooling, 3 ml of toluene are added to each tube and mixed to obtain two phases. The upper phase is collected, and the optical density (OD) of this phase is measured using a spectrophotometer (Shimadzu UV-1600, Kyoto, Japan) at a wavelength of 520 nm.

### Soluble sugars measurement

The Anthrone method was used to measure soluble sugars (glucose and fructose)^72^. Soluble sugars (glucose and fructose) are extracted by macerating barley leaves in 2 mL of 80% ethanol, followed by incubation in a water bath at 75 °C for 40 min and centrifugation at 10,000*g* for 5 min at 4 °C. The supernatant was then collected, and the procedure was repeated a second time with the pellet. The two obtained supernatants (total volume = 4 mL) from different samples were placed in glass screw-cap tubes. These tubes were placed in a vortex evaporator (Buchler) to evaporate the alcohol. In each tube, the residue was diluted in 10 mL of distilled water (analytical solution). The Anthrone reagent (SIGMA) was prepared at a concentration of 2 g/L in 75% sulfuric acid. To 1 mL of the sample (0.25 mL of the analytical solution + 0.75 mL of distilled water), 4 mL of Anthrone reagent was added. The blend was vortexed and then placed in a heated bath at 100 °C for 8 min. Subsequently, the tubes were cooled in an ice bath, and the absorbance was measured at 625 nm using a Shimadzu UV-1600 spectrophotometer (Kyoto, Japan).

### Malondialdehyde measurement (MDA assay)

Lipid peroxidation was indirectly assessed by quantifying the levels of malondialdehyde using the method described by^73^. To perform the assay, 100 mg of plant material was ground and then homogenized in 2 ml of 0.1% trichloroacetic acid (TCA). The homogenate is centrifuged at 10,000*g* for 15 min at 4 °C. Next, 0.5 ml of the supernatant is mixed with 1.5 ml of 0.5% thiobarbituric acid (prepared in 20% TCA) and incubated at 90 °C for 20 min. After stopping the reaction in an ice bath, the samples are centrifuged at 10,000*g* for 5 min. The absorbance of the supernatant is read at 532 nm. The optical density is then corrected by subtracting the non-specific absorbance at 600 nm. The MDA content, expressed in nmol MDA per gram of fresh weight (nmol MDA g^−1^ FW), is determined using its extinction coefficient of 155 mM^−1^ cm^−1^.

### Data analysis

One-way ANOVA was used to evaluate the variables from the treatments. After obtaining significant results (p < 0.05), multiple comparisons using the Tukey HSD test were applied to identify significant differences between treatments. All analyses were performed Utilizing the Stats package (R Core Team, (V0.1.0, 2019). Using the Ward approach, we performed a hierarchical cluster analysis of the matrix of correlations among the obtained data. Correlation analysis and Heatmap were conducted, respectively, following the Pearson’s correlation coefficient method, using “corrplot (V0.92, 2021)” and “METAN (V1.18.0, 2023)” packages in R. For ranking the barley genotypes under salinity stress, associated traits were considered in traits modelling (stepwise regression approach) to derive the response equation using statistical software (R Core Team, (V0.1.0, 2019).

### Plant guideline statement

The study complies with relevant institutional, national, and international plant guidelines and legislation. Authors have the permission to use the seeds of the plant materiel mentioned in “Material and methods” section.

## Conclusion

Salinity tolerance is a complex phenomenon as it requires the combination of different traits. In order to develop a salt tolerant germplasm, we need to focus on studying response mechanisms face to high salt stress. Our study focused on differentiating barley genotypes based on several physiological and biochemical traits. We can conclude that barley genotypes respond differently to salt stress. MDA, proline and soluble sugars increased and are more important in Ardhaoui than all other genotypes specifically Lemsi grown under saline conditions (T1 and T2). Photosynthetic activity is more important in Ardhaoui and Rihane than the other genotypes in all treatments. These results will guide future research in focusing on integrative stress management strategies and validating and applying them in real-world agricultural conditions.

### Supplementary Information

Supplementary Table S1.

Supplementary Table S2.

## Data Availability

The datasets used and analysed during the current study available from the corresponding author on reasonable request.
